# Editor's Message

**DOI:** 10.29173/jchla29684

**Published:** 2023-04-01

**Authors:** Pawliuk Colleen

**Affiliations:** Rédactrice en chef, JABSC / JCHLA

Welcome to the JCHLA/JABSC Spring Issue! This issue we have five book reviews on topics related to equity, diversity and inclusion, online instruction, misinformation and more. We also have a product review on Elicit, a free artificial intelligence tool that can expedite the evidence synthesis process.

Also, in this issue we have two announcements for our readers. Registration for the CHLA/ABSC 2023 Conference is now open. If you are submitting your work to the CHLA/ABSC conference, we also invite you to consider submitting to JCHLA/JABSC. Writing up your research article or program description into a full article provides important detail and context for those interested in your work. Additionally, we are pleased to announce a Special Issue in April 2024 on the challenges and innovations of health information professionals during the COVID-19 pandemic. You can read more our requirements for any potential submissions in our Author Guidelines.

**Colleen Pawliuk**

*JCHLA/JABSC Editor-in-Chief*

*Email:* editor@chla-absc.ca

Bienvenue au numéro de printemps du JABSC / JCHLA ! Ce numéro contient cinq critiques de livres sur des sujets liés à l'équité, à la diversité et à l'inclusion, à l'enseignement en ligne, à la désinformation et plus encore. Nous avons également une revue de produit sur Elicit, un outil d'intelligence artificielle gratuit qui peut accélérer le processus de synthèse des connaissances.

Dans ce numéro, nous avons également deux annonces à faire à nos lecteurs. L'inscription au congrès ABSC / CHLA 2023 est désormais ouverte. Si vous soumettez votre recherche au congrès de l’ABSC / CHLA, nous vous invitons également à envisager de la soumettre au JABSC / JCHLA. La rédaction de votre article de recherche ou votre description de programme dans un article complet fournit des détails et un contexte importants pour ceux et celles qui s'intéressent à votre travail. En outre, nous avons le plaisir d'annoncer un numéro spécial en avril 2024 sur les défis et les innovations des professionnels-les de l'information sur la santé pendant la pandémie COVID-19. Pour en savoir plus sur nos exigences en matière de soumission d'articles, consultez nos Directives aux auteurs-es.

**Colleen Pawliuk**

*Rédactrice en chef, JABSC / JCHLA*

*Courriel:* editor@chla-absc.ca

